# Does diabetes mellitus increase the incidence of early thrombosis in deep vein following unicompartmental knee arthroplasty: a retrospective cohort study

**DOI:** 10.1186/s12877-022-03153-w

**Published:** 2022-05-24

**Authors:** Xufeng Jiao, Zheng Li, Shuai An, Jiang Huang, Mingli Feng, Guanglei Cao

**Affiliations:** Department of Orthopedics, Xuanwu Hospital Capital Medical University, Beijing, 100053 China

**Keywords:** Diabetes mellitus, Deep vein thrombosis, Glycosylated hemoglobin, Unicompartmental knee arthroplasty

## Abstract

**Background:**

Many patients following unicompartmental knee arthroplasty (UKA) also suffer from diabetes mellitus, which may lead to an increased likelihood of postoperative deep venous thrombosis (DVT). Therefore, we evaluated whether DVT incidence would increase 3 days following UKA in diabetic patients.

**Methods:**

Patients who underwent UKA from August 2018 to September 2021 in our hospital were retrospectively included. Age, gender, body mass index, hypertension, mode of anesthesia, surgery time, intraoperative blood loss, tourniquet pressure and time, and glycosylated hemoglobin concentration were recorded as confounders. We compared the incidence and type of DVT between non-diabetic and diabetic patients and evaluated the effect of glycosylated hemoglobin levels on DVT.

**Results:**

Of all the 224 patients, 96 had diabetes and 128 did not. Within 3 days after surgery, DVT occurred in 25 cases in the diabetic group and 17 cases in the non-diabetic group (*p* < 0.05), and the difference mainly exists in the lower limbs on the surgical side. Logistic regression analysis demonstrated that the risk of DVT in the diabetic group was 4.50 times higher compared with the non-diabetic group. For every 1 unit increase of glycosylated hemoglobin, the incidence of DVT increased 2.35 times. Differences in age, gender, body mass index, hypertension, mode of anesthesia, surgery time, intraoperative blood loss, tourniquet pressure, and time between the two groups were not significant.

**Conclusions:**

The incidence of DVT in diabetic patients within 3 days after UKA was significantly higher than that in non-diabetic patients and was proportional to the concentration of glycosylated hemoglobin.

## Introduction

Orthopedic surgery, especially hip or knee arthroplasty, can remarkably increase the risk of hospital-acquired deep vein thrombosis (DVT) [1]. Even with prophylactic antithrombotic therapy, the incidence of DVT after total joint arthroplasty is close to 20% [2].

The occurrence of DVT is related to many aspects, such as gender, age, anesthetic mode, body mass index (BMI), heart disease, diabetes mellitus, active cancer, and so on [3]. The global population is entering an aging stage, followed by an increasing proportion of patients with diabetes in arthroplasties. Researchers have confirmed that when patients suffer from diabetes, their coagulation system [4], hemostatic system [5], and fibrinolysis [6] are abnormal, resulting in the procoagulant and thrombotic susceptibility, so diabetes may increase the risk of DVT [7, 8]. A retrospective study has shown that diabetic patients had a 2.71 times higher risk of postoperative DVT than that of non-diabetic patients following total knee arthroplasty (TKA) [9].

Unicompartmental knee arthroplasty (UKA) has become widely used in recent years. Compared with TKA, UKA is characterized by less trauma, short surgery time, and rapid postoperative recovery [10, 11]. These advantages may lead to a lower postoperative incidence of DVT in UKA than in TKA, making UKA the first choice of many doctors for end-stage unicompartmental knee osteoarthritis [12]. To our knowledge, the effect of diabetes on postoperative DVT has not been studied in UKA. Therefore, we performed a retrospective cohort study to compare the risk of DVT following UKA between diabetic and non-diabetic patients. The hypothesis is that diabetes increases the postoperative DVT risk in patients with UKA. And the risk is related to the glycosylated hemoglobin concentration.

## Methods

### Inclusion and exclusion criteria

This retrospective cohort study included the patients who underwent unilateral UKA with diagnosis of knee osteoarthritis from August 2018 to September 2021 in our hospital. We excluded patients who had infectious, gouty, or rheumatoid arthritis, DVT revealed by preoperative ultrasonography, previous history of DVT, or vascular surgery in lower extremities. Patients with coronary heart disease, arrhythmia, chronic heart failure, tumor history, long-term prophylactic use of anticoagulants such as aspirin, or severe postoperative complications were also excluded. The patients were assigned to the diabetes group and non-diabetes group, respectively.

### Surgery technique

No prophylactic antithrombotic therapy was given before surgery. The anesthesiologist chose general or intraspinal anesthesia depending on the patient’s condition. A tourniquet was used from the beginning of the surgery until the prosthesis was fixed with bone cement, and the pressure of the tourniquet was generally set as systolic pressure plus 100 mmHg. The surgeries were performed with medial parapatellar approach incision, cemented Oxford phase III medial UKA prosthesis (two pegs; MP instrument; Biomet UK LTD, Waterton Industrial Estate, Bridgend CF31 3XS, UK), and cocktail injection (tranexamic acid 60 ml, parecoxib sodium 40 mg, epinephrine 0.15 ml, ropivacaine hydrochloride 20 ml:200 mg, oxycodone 10 ml:1 mg, injected into the joint capsule, synovium, subperiosteum, and subcutaneous tissues).

### Postoperative management

No drainage was used after surgery. Ankle flexion and quadriceps muscle contraction exercises were required as early as possible. Full weight-bearing ambulation with crutches should be conducted four hours postoperatively. The lower extremity pneumatic circulation pump was used on the night of the surgery and was stopped the next morning. Diabetics controlled their fasting and postprandial blood glucose concentrations to 5-7 mmol/L and 8–10 mmol/L, respectively, by using long-acting insulin at bedtime and short-acting insulin before meals.

### DVT diagnosis and treatment

To avoid the risk of early-stage bleeding, on the third day postoperatively, deep vein color Duplex sonography was performed and prophylactic or therapeutic dose of anticoagulant was used. Two experienced sonographers performed deep vein color Duplex sonography of lower extremities for all patients. If there was a solid echo in the lumen and after the probe was pressurized, the vascular lumen was not compressed, and no obvious blood flow signal was seen in Color Doppler flow imaging, it can be diagnosed as DVT. When the diagnosis results of the two sonographers were inconsistent, a unified final result will be given after consultation. Based on the ultrasound results, DVT was diagnosed and classified into distal (intermuscular vein thrombosis of the calf) and proximal (thrombosis in and above the popliteal vein) types, and then the therapeutic dose of low molecular weight heparin (LMWH; Enoxaparin Sodium Injection, 0.4 ml: 4,000 AxaIU) was given. Other patients without DVT underwent prophylactic anticoagulant therapy with LMWH (Enoxaparin Sodium Injection, 0.2 ml: 2,000 AxaIU) to prevent thrombosis. The patients received LMWH for 3–5 days in hospital and oral rivaroxaban for two weeks after discharge.

### Statistical analysis

Demographics data included BMI, age, gender, surgery side, and concomitant disease. Recorded glycosylated hemoglobin value (normal value: 4%-6%), intraoperative tourniquet pressure and duration, and intraoperative blood loss.

*t*-test was used to compare continuous variables, and Pearson Chi-square test was used to compare counting variables. Logistic regression model was performed on the factors that may affect the incidence of DVT, and the odds ratio (OR) and its 95% confidence interval (CI) were calculated. To eliminate multicollinearity, diabetes and glycosylated hemoglobin were included in the regression model separately. Statistical packages R (The R Foundation; http://www.r-project.org; version 3.4.3) and Empower (R) (www.empowerstats.com, X&Y solutions, inc. Boston, Massachusetts) were used for analysis. If *p*-value < 0.05, the difference was considered significant.

## Results

Two hundred eighty-eight patients underwent UKA from August 2018 to September 2021 in our hospital. Among them, 64 patients were excluded according to the exclusion criteria (2 patients of DVT showed by preoperative ultrasound, 1 patient of colon cancer history, 42 patients of coronary heart disease, 16 patients of long-term use of aspirin, and 3 patients of lower extremity surgery history). 25 of the 64 patients excluded had diabetes. A total of 224 patients were finally enrolled, 96 of whom had diabetes mellitus. No significant difference was observed between the two groups except for glycosylated hemoglobin (*p* < 0.001) and DVT (*p* < 0.05). Table 1 gives the characteristics of the study population.

**Table 1:** Clinical features and population distribution

	Diabetes mellitus	Non-diabetes mellitus	*P*-value
Number	96	128	
Age	71.1 ± 5.9	68.0 ± 3.7	0.472
BMI (kg/m.^2^)	27.3 ± 3.5	26.9 ± 3.1	0.634
Gender			0.491
Male	22 (22.9%)	47 (36.7%)	
Female	74 (77.1%)	81 (63.3%)	
Glycosylated hemoglobin	6.6 ± 1.2	5.4 ± 0.7	< 0.001
Hypertension	77 (80.2%)	97 (71.1%)	0.363
Anesthesia			0.716
General anesthesia	26 (27.1%)	39 (30.5%)	
Intravertebral anesthesia	70 (72.9%)	89 (69.5%)	
Duration of surgery (min)	102.5 ± 18.3	101.3 ± 16.8	0.857
Duration of tourniquet (min)	65.2 ± 9.2	66.1 ± 8.7	0.526
Tension of tourniquet (mmHg)	262.1 ± 5.5	260.7 ± 3.6	0.695
Intra-operative blood loss (ml)	50.8 ± 17.3	47.2 ± 12.2	0.453
DVT	25 (26.0%)	17 (13.3%)	< 0.05

Data: Mean ± SD / *N*

*BMI* Body mass index, *DVT* Deep venous thrombosis

**Fig. 1:**
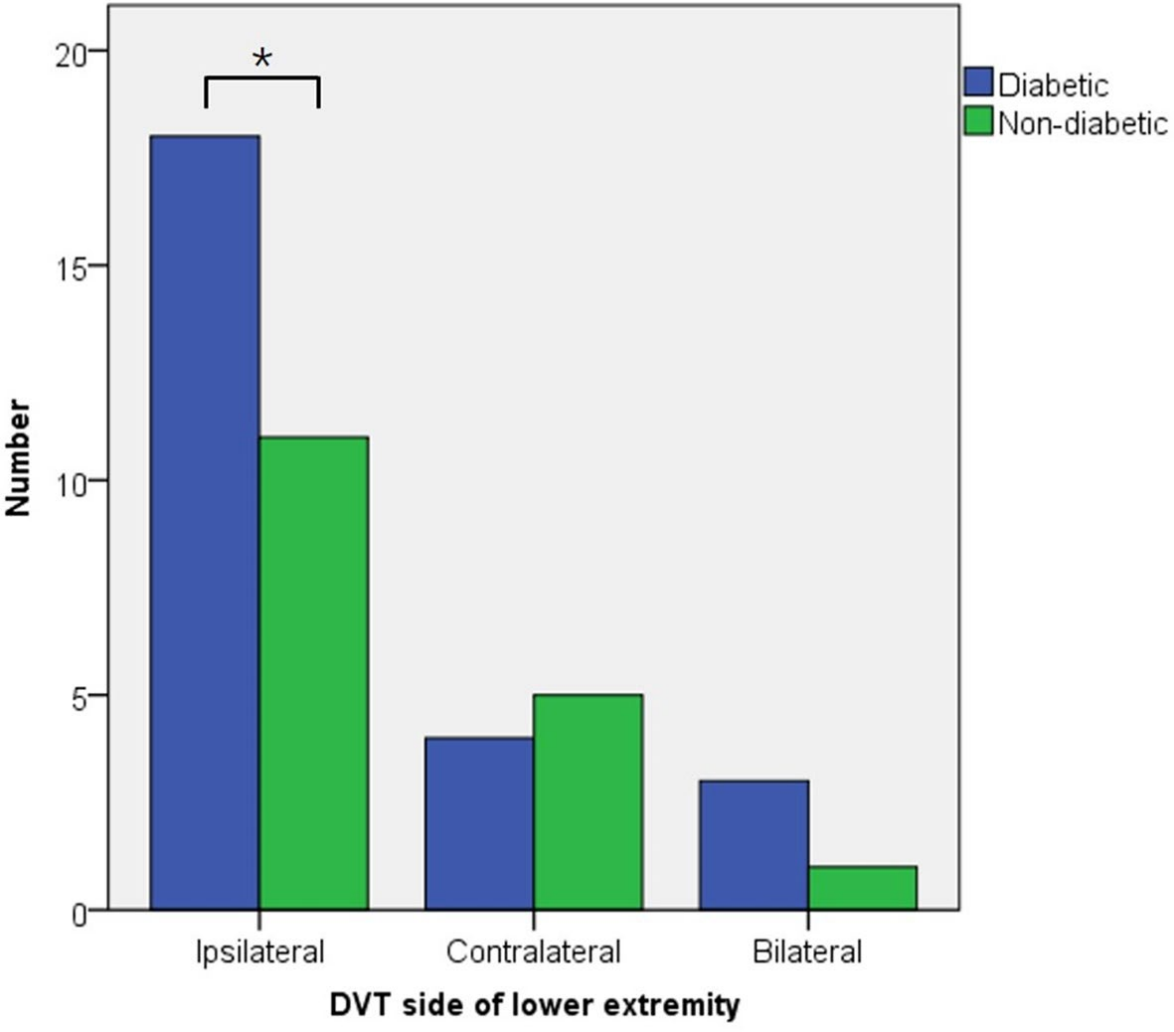
Bar graph of deep vein thrombosis (DVT) side distribution in lower limbs after unicompartmental knee arthroplasty in diabetic and non-diabetic patients. There was a significant difference in DVT incidence in the lower limbs on the surgical side between the two groups. (*: *p* < 0.05)

On the third day after surgery, color Doppler ultrasound showed distal DVT in 42 patients. All DVTs were distal type and asymptomatic. The incidence of DVT in the diabetic group (26.0%) was higher than that in non-diabetic group (13.3%, *p* < 0.05). Among those with diabetes, 18 appeared in the ipsilateral leg, 4 in the contralateral leg, and 3 in the bilateral legs. Among those non-diabetic patients with DVT, 11 appeared in the ipsilateral leg, 5 in the contralateral leg, and 1 in bilateral legs. There was a significant difference in the incidence of DVT in the operative leg between the two groups (Fig. 1). After adjusting for BMI, age, hypertension, and some other confounding factors, multivariate logistic regression model showed that diabetes mellitus was a risk factor of DVT. Patients with diabetes had a 4.498 times greater risk of developing DVT than those without diabetes (Table 2). The distribution of glycosylated hemoglobin level of patients is shown in Table 3. Substituting the glycosylated hemoglobin value for diabetes and performing multiple regression analysis again, the results showed that the risk of developing DVT increased 2.351-fold (95% CI: 1.285–5.952, *p*< 0.05) for every unit of glycosylated hemoglobin increased.

**Table 2:** Multivariate Logistic regression analysis of influencing factors of DVT

Factor	OR	95% CI	*P*
Age	0.980	0.916–1.129	0.563
Gender	1.952	0.348–22.422	0.896
BMI (kg/m.^2^)	1.263	0.118–5.569	0.872
Hypertension	1.684	0.085–3.236	0.433
Diabetes mellitus	4.498	2.658–9.256	< 0.05
Tension of tourniquet (mmHg)	1.632	1.167–2.561	0.389
Duration of tourniquet (min)	1.022	0.899–1.144	0.481
Duration of surgery (min)	1.238	0.953–1.392	0.658
Intra-operative blood loss (ml)	0.856	0.778–0.973	0.784

*BMI* Body mass index

**Table 3:** The distribution of glycosylated hemoglobin level

Glycated hemoglobin level	5.0–5.9%	6.0–6.9%	7.0–7.9%	8.0–8.9%	≥ 9.0%
Number	131	77	8	5	3

## Discussion

In this retrospective cohort study, we found that the incidence of DVT in early-stage after UKA was closely associated with diabetes, and was proportional to glycosylated hemoglobin levels.

DVT may cause pulmonary embolism (PE). The highest risk of symptomatic PE occurs within the first week postoperatively, with 81% occurring within three days after the surgery [13, 14]. Therefore, it is very important to prevent the occurrence of DVT and PE in the early stage after arthroplasty. DVT is caused by multiple factors, including BMI and increasing age, major surgery, congestive heart failure, arrhythmia, active cancer and, dyskinesia [15, 16]. Diabetes is also a potential risk factor for an increased incidence of DVT. Petrauskiene’s study found that after adjusting for age, the venous thromboembolism (VTE) risk in patients with diabetes was more than twice of that in non-diabetic people [7]. After further adjustment for race and gender, Tsai suggested that the risk of VTE at baseline in diabetic patients was 1.70 times higher compared with those who had normal fasting blood glucose (95% CI, 1.20–2.40) [17]. Retrospective analysis and prospective evaluation of renal transplants also showed that diabetes was remarkably related to VTE [8, 17, 18].

Our study found that patients with diabetes had a significantly higher incidence of DVT after UKA. UKA damages soft tissue and bone, leading to release of tissue factor that initiates the coagulation cascade [19]. Surgical trauma can also lead to vascular endothelial injury, promote thrombotic responses, and enhance blood coagulation [20]. Decreased mobility during postoperative recovery may cause venous stasis. When patients have diabetes mellitus, their hemodynamic abnormalities are aggravated. The platelet has higher adhesion levels and is more likely to aggregate [5]. Therefore, venous stasis, vascular endothelial dysfunction, and blood hypercoagulability under the combined effect of surgery attack and diabetes may exacerbate the development of DVT [21]. Intraoperative use of tourniquets hinders blood flow and aggravates blood stasis [22].This explains why the difference in the incidence of DVT mainly exists in the lower extremities on the surgical side. In addition, in our study, all DVTs were distal type. Some studies also found that the incidence of distal DVT after orthopedic surgery was significantly higher than that of the proximal [23, 24]. This may be related to the greater effect of tourniquet on venous blood stasis in distal limbs [25]. Asymptomatic distal DVT is generally considered to be self-limiting [26]. However, studies have found that when distal DVT is not treated effectively in time, the probability of distal DVT extending proximally is as high as 20% [27]. Proximal extension can lead to serious embolic complications, including PE. Therefore, asymptomatic distal DVT also requires attentive observation and effective prophylactics to prevent proximal extension of the thrombus. In our study, there was a small percentage of patients with contralateral or bilateral DVT after surgery in both diabetic group and non-diabetic group. Although the proportion is low, the surgeon should not ignore the possibility of postoperative DVT in the non-operative lower limbs.

A previous retrospective study of 358 knees showed that DVT incidence within 14 days after TKA in patients who had diabetes mellitus was 2.7 times higher compared with those without diabetes mellitus [9]. This is consistent with our results. Compared with TKA, both the surgery time of UKA and the interval between surgery and ambulation are much shorter. Thus, the duration of applying tourniquet, the amount of blood loss during surgery, and immobilization of the affected limb have less influence on the results, which resulted in the lower incidence of DVT in our study (18.8%) than that of TKA (55.3%) [9]. In addition, no patient had thrombosis-related symptoms such as lower extremity edema, severe pain, and dyspnea in our study. This is also the potential advantage of UKA.

Glycosylated hemoglobin reflects the average plasma glucose concentration over a period and is recommended for blood glucose control in those who had diabetes. Therefore, we further investigated quantitatively whether glycosylated hemoglobin levels in diabetic patients were associated with the incidence of DVT. The result showed that for every 1 unit increase in glycosylated hemoglobin, the risk of DVT increased by 2.351 times (95% CI: 1.285–5.952, p < 0.05). Therefore, when diabetic patients have poor glucose level control, the risk of DVT after surgery will increase sharply. However, the mechanism of glycosylated hemoglobin’s effect on macrovascular disease is not clear [28].

There are several limitations in our study. First, venography is the gold standard for diagnosing DVT, but its invasiveness and expensiveness limit its application. Color Doppler ultrasound is the most commonly-used preliminary screening method for DVT in clinical practice. A prospective clinical study comparing the accuracy of ultrasound versus venography for the diagnosis of DVT after TKA showed that the specificity and sensitivity of ultrasound were 63% and 87%, respectively, when venography results were used as a baseline reference. Therefore, as a non-invasive, safe, and convenient inspection method for DVT after arthroplasty, ultrasound is a reliable tool for preliminary screening of clinically suspected DVT [25]. Especially for proximal DVT, doppler ultrasonography provides good sensitivity (86%) and specificity (100%) [29]. The same patient was evaluated by two sonographers in our study, so the interference of ultrasound errors to the results can be ignored. Second, different types of diabetes may have different effects on DVT. However, since the admission records did not distinguish the type of diabetes, the influence of the type on DVT needs to be further studied. Third, different anesthetic methods exert different influences on hemodynamics, thus affecting the occurrence of postoperative DVT [30]. In our study, all patients received general or intraspinal anesthesia, and no significant difference was observed between them (*p* = 0.716). Additionally, the incidence of DVT was the highest in the first week following surgery [31]. However, patients who underwent UKA can be discharged from the hospital about five days after surgery due to the minimal trauma and rapid recovery, and VTE mainly occurs within three days after surgery [13, 14], so color Doppler ultrasound data were collected three days after surgery.

## Conclusions

The risk of developing DVT in the early stage after UKA was significantly increased in the patients with diabetes mellitus. A higher level of glycosylated hemoglobin was related to a higher risk of DVT. It is hoped that a large-scale prospective population-based study in the future, including a detailed description of diabetes typing and glucose tolerance, will help to further clarify the relationship between diabetes and DVT.

## Data Availability

The datasets used and analyzed during the current study are available from the corresponding author on reasonable request.

## Abbreviations

DVT: Deep vein thrombosis
BMI: Body mass index
UKA: Unicompartmental knee arthroplasty
TKA: Total knee arthroplasty
PE: Pulmonary embolism
VTE: Venous thromboembolism
